# Si-rich SiN_x_ based Kerr switch enables optical data conversion up to 12 Gbit/s

**DOI:** 10.1038/srep09611

**Published:** 2015-04-29

**Authors:** Gong-Ru Lin, Sheng-Pin Su, Chung-Lun Wu, Yung-Hsiang Lin, Bo-Ji Huang, Huai-Yung Wang, Cheng-Ting Tsai, Chih-I Wu, Yu-Chieh Chi

**Affiliations:** 1 Graduate Institute of Photonics and Optoelectronics, Department of Electrical Engineering, National Taiwan University (NTU), No.1, Sec. 4, Roosevelt Road, Taipei 106, Taiwan, Republic of China

## Abstract

Silicon photonic interconnection on chip is the emerging issue for next-generation integrated circuits. With the Si-rich SiN_x_ micro-ring based optical Kerr switch, we demonstrate for the first time the wavelength and format conversion of optical on-off-keying data with a bit-rate of 12 Gbit/s. The field-resonant nonlinear Kerr effect enhances the transient refractive index change when coupling the optical data-stream into the micro-ring through the bus waveguide. This effectively red-shifts the notched dip wavelength to cause the format preserved or inversed conversion of data carried by the on-resonant or off-resonant probe, respectively. The Si quantum dots doped Si-rich SiN_x_ strengthens its nonlinear Kerr coefficient by two-orders of magnitude higher than that of bulk Si or Si_3_N_4_. The wavelength-converted and cross-amplitude-modulated probe data-stream at up to 12-Gbit/s through the Si-rich SiN_x_ micro-ring with penalty of −7 dB on transmission has shown very promising applicability to all-optical communication networks.

Benefiting from the mature fabrication technology in the Si-based VLSI industry, versatile all-Si based active and passive optical devices including modulators, switches, couplers and light emitters have been fabricated to meet the demands of next-generation optoelectronic integrated circuits (OEIC)^1^,^2^,^3^,^4^. Since the first observation of optical gain on Si quantum dot doped SiO_x_ (SiO_x_:Si-QD) by Pavesi *et al.* in 2000^5^, the nano-scale Si doped dielectric or semiconductor matrices have been comprehensively studied for mandatory applications of serving as light emitting diodes^6^,^7^,^8^,^9^,^10^,^11^, distributed Bragg reflectors^12^,^13^, waveguide amplifiers^14^, etc., in Si based OEICs. Later on, the Si-rich SiO_x_ based near-infrared electroluminescent diode^15^, and the Si-QD doped SiO_x_ waveguide based all-optical switches and modulators^16^,^17^,^18^,^19^ employing free-carrier absorption (FCA) effect were also demonstrated. However, the relatively long free-carrier lifetime (~1 ns for bulk Si and 0.01–10 μs for Si-QD) limits the switching speed of these devices^17^. To achieve high-speed data transmission, the Si-QD based photonic devices using nonlinear effects such as two-photon-absorption (TPA) and third-order Kerr effects are considered^20^. However, the TPA based approach in bulk Si or Si-QDs suffers from two distinct drawbacks including broadband absorption and lifetime limited switching^17^,^21^, and the Kerr switching in bulk Si is usually degraded by high TPA phenomenon^22^,^23^,^24^. Recently, the all-optical Kerr switching was demonstrated by sandwiched Si/SiO_x_:Si-QD/Si slot waveguide with high coupling loss^22^.

Apparently, the materials with negligible TPA but huge Kerr coefficient are preferred, and the silicon nitride (Si_3_N_4_) ring waveguide is subsequently considered as a nice candidate to achieve the nonlinear all-optical switching^25^. With its refractive index larger than that of SiO_2_ matrix^26^ and its bandgap energy higher than that of Si^27^,^28^, the Si-rich SiN_x_ enables small-dimension channel waveguide without using slot structure, which favors the sole Kerr nonlinear process with almost negligible TPA. Nevertheless, the nonlinear property of the stoichiometric Si_3_N_4_ is usually ten times smaller than that of bulk Si^22^,^23^. The nano-scale Si-QD doped Si_3_N_4_ film is an alternative to improve nonlinearity of the Si_3_N_4_ film, which reveals much larger magnitude on the real part of third-order nonlinearity coefficient than that ever observed in single- or poly-crystalline Si^29^. Even after doping with the Si-QDs, the reduced bandgap energy of the Si-rich SiN_x_ is still high enough to suppress the TPA effect.

To meet the demand of next-generation integrated circuit for photonic interconnection with micro-ring based cross-wavelength data converter and format inverter, the all-optical Kerr switching in a Si-rich SiN_x_ channel ring waveguide with non-return-to-zero on-off keying (NRZ-OOK) up to 12 Gbit/s is demonstrated for the first time. With optical pumping data-stream, the Kerr nonlinearity induces a wavelength red-shift on the transfer function of the Si-rich SiN_x_ based micro-ring, which eventually leads to cross-amplitude modulation on the probe beam at a wavelength coincident with one of the notch points at the throughput transfer function. The ultrafast Kerr effect induced by the input optical data stream instantly modifies the nonlinear refractive index of the Si-rich SiN_x_ to provide high-speed cross-wavelength optical data conversion up to 12 Gbit/s.

## Results

### Structural and Compositional Characteristics of Si-rich SiN_x_ Channel/Ring Waveguide

**Figure 1:**
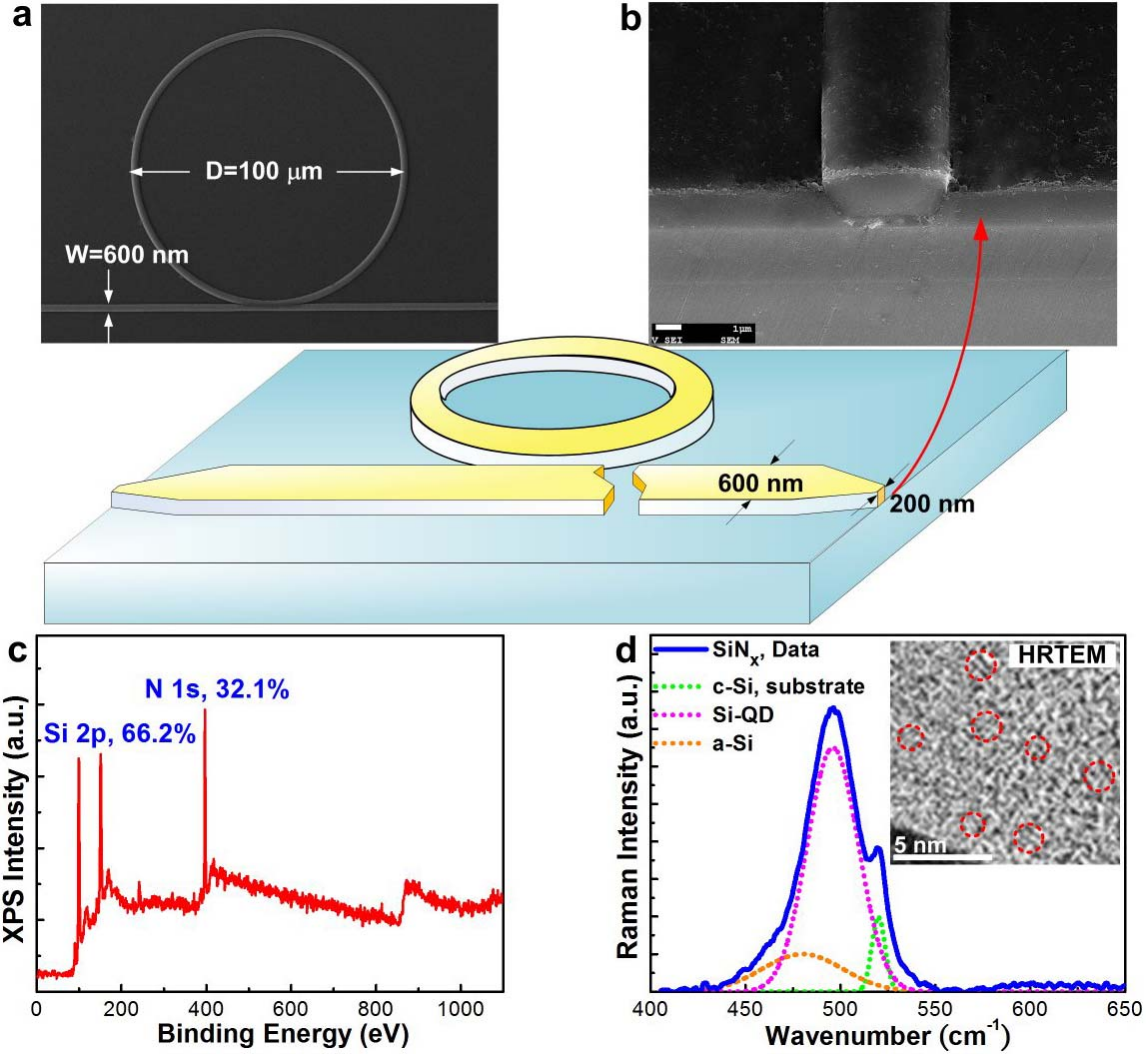
The configuration of the Si-rich SiN_x_ waveguide based nonlinear Kerr switch and the structural analyses of Si-rich SiN_x_. (a) The optical microscopy (OM) image and (b) the scanning electron microscopy (SEM) image of the Si-rich SiN_x_ waveguide based nonlinear Kerr switch. (c) The full-band XPS spectrum of the Si-rich SiN_x_ film with relative Si and N composition ratios. (d) The Raman scattering spectrum of the Si-rich SiN_x_ film with decomposed functions related to a-Si, Si-QD, and c-Si components. Inset: the HRTEM cross-section-view image of the Si-rich SiN_x_ film.

The configuration of the Si-rich SiN_x_ waveguide based nonlinear Kerr switch is shown in Fig. 1a, where the height, width and length of the Si-rich SiN_x_ channel waveguide are defined as 400 nm, 600 nm and 3 μm, respectively. To enhance the coupling efficiency by 2-dB/facet, the inversed taper is employed with its waveguide width gradually increasing from 200 to 600 nm at both sides within a tapered length of 200 μm, as shown in Fig. 1b. For compositional analysis, the Si-rich SiN_x_ layer with a thickness of 400 nm was synthesized on a 3-μm thick thermal SiO_2_ on Si substrate. The X-ray photoelectron (XPS) microscopy of the Si-rich SiN_x_ shown in Fig. 1c reveals the Si and N atomic concentrations of 66.2% and 32.1%, respectively, corresponding to the excessive Si concentration of 23.4% in the Si-rich SiN_x_. Three different components are decomposed from Raman scattering spectrum at 450–500 cm^−1^ in Fig. 1d, as contributed by the single crystalline Si substrate peak at 520 cm^−1^ with the linewidth of 6 cm^−1^, the Si-QD related peak at 495 cm^−1^ with the linewidth of 21 cm^−1^, and the amorphous Si related peak at 480 cm^−1^ with the broadest linewidth of 31 cm^−1^ 30. The Si-QD size of 0.9 ± 0.1 nm estimated from the Raman scattering spectrum shows good agreement with the high-resolution transmission electron microscopic (HRTEM) analysis.

### TPA-free throughput response and nonlinear Kerr switching analysis

**Figure 2:**
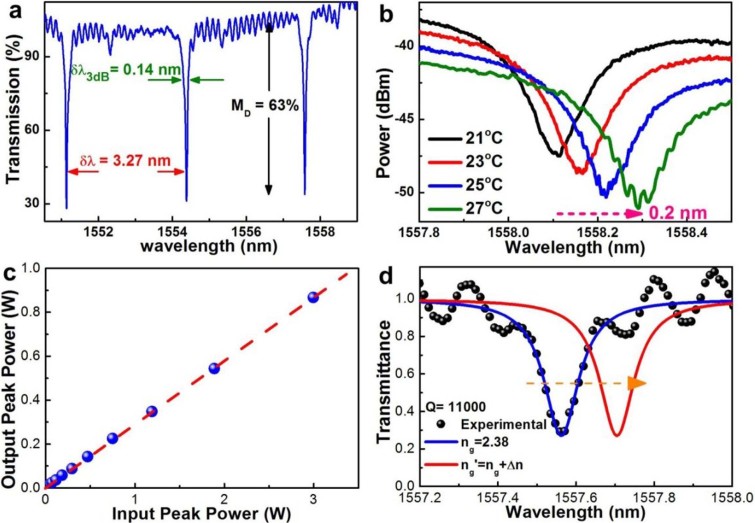
The configuration of the Si-rich SiN_x_ waveguide based nonlinear Kerr switch with its notched transmittances at different temperatures. (a) The Si-rich SiN_x_ micro-ring based transmission spectrum. (b) The notched transmittances of the Si-rich SiN_x_ micro-ring waveguide at different temperatures. (c) The peak transmitted power of the Si-rich SiN_x_ based channel waveguide vs. the incident peak power at a wavelength of 1550 nm. (d) The simulated wavelength shift on the probe transmission spectra before and after pumping the micro-ring waveguide resonator.

**Figure 3:**
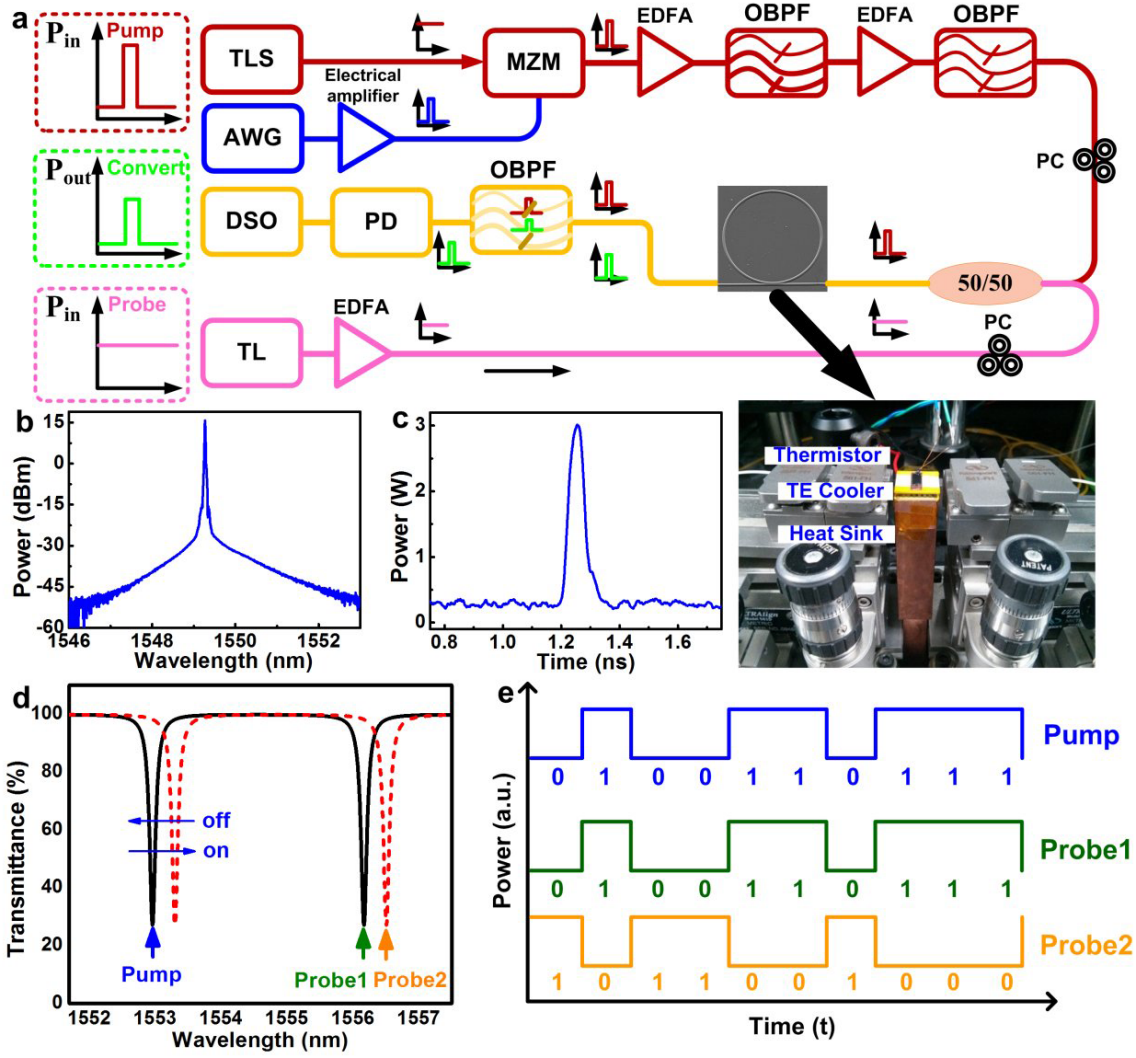
Experimental setup of the pump-probe system with the Kerr effect induced notch red-shift and cross-amplitude modulation. (a) The experimental setup of the pump-probe system using an externally modulated RZ-OOK data-stream based pump beam and a continue-wave signal-mode laser based probe beam. (b) The optical spectrum of the externally modulated optical pump signal. (c) The time-domain trace of the single “on-bit” data within externally modulated optical RZ-OOK data-stream based pump signal. (d) The illustration of the nonlinear optical Kerr effect induced shift on the notched resonant dip and (e) the corresponding cross-amplitude modulation on the probe signal passing through the rib/micro-ring waveguide resonator based all-optical Kerr switch.

The Si-rich SiN_x_ micro-ring based transmission spectrum exhibits periodically notched dip with a spacing of *δν* = 305.8 THz (*δλ* = 3.27 nm), where its transmittance drops by nearly 70% within a full-width-at-half-maximum (FWHM) of *δλ*_3dB_ = 0.14 nm to cause a Q-factor^31^ of 1.1 × 10^4^, as shown in Fig. 2a. When increasing the temperature of Si-rich SiN_x_ based micro-ring, the refractive index of the SiN_x_ with a positive *dn*/*dT* is increased due to the thermal-optics effect^32^. In experiment, the transmission dip of the SiN_x_ micro-ring waveguide is up-shifted by 0.21 nm when increasing the temperature from 21°C to 27°C, as shown in Fig. 2b. Owing to the large drifting slope of 0.35 nm/°C, the temperature stabilization at 23 ± 0.1°C is performed by a TE cooler in connection with a copper based heat sink via a thermistor feedback, as shown in Fig. 3a. Whether the TPA phenomenon exists or not plays an important role on the nonlinear Kerr switching efficiency^33^. To rule out the existence of the TPA phenomenon in the Si-rich SiN_x_ channel waveguide, a direct measurement for obtaining the throughput linearity of the Si-rich SiN_x_ channel waveguide. As shown in the Fig. 2c, the transmitted peak power arises linearly by increasing the incident peak power from 1 mW to 3 W. There is no nonlinear throughput as well as TPA phenomenon happened in the Si-rich SiN_x_ based channel waveguide even under intense pumping. This is attributed to the extremely large bandgap energy of up to 3 eV for the Si-rich SiN_x_^34^. When comparing with the bulk Si based waveguide devices with the inherently strong TPA effect that dominates over the nonlinear optical Kerr effect at the optical telecommunication wavelengths, the Si-rich SiN_x_ is undoubtedly more suitable than the bulk Si to serve as the waveguide material of the nonlinear all-optical Kerr switch.

By pumping the Si-rich SiN_x_ micro-ring resonator with high-power optical data-stream, the wavelength shift of the notched resonance can be attributed to a group index change. The red-shifted transmission function can be described by Ref. 35,
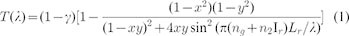


Therefore, the nonlinear refractive index (*n_2_*) can be estimated by using *n_2_* *=* *Δn/I_r_*, where *I_r_* represents the enhanced peak intensity inside the Si-rich SiN_x_ micro-ring resonator at the resonant wavelength, as defined by *I_r_* *=* *M* × *I_pump_* with the magnification factor *M* derived as^36^,
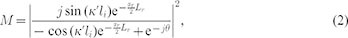
where *α_r_* is the absorption coefficient of the micro-ring resonator, *κ*′ is the coupling coefficient between two directional waveguides. The *l_i_*, *L_r_* and *θ* represent the interaction length, the circumference of ring resonator and the phase-shift, respectively. After obtaining the red-shift of transmission spectrum by pumping the micro-ring waveguide resonator as shown in Fig. 2d, the nonlinear refractive index can be numerically simulated by the equation (1) with structural and material parameters listed in Table 1. Note that in the above calculation, the group index is set to be the same as the refractive index.

### Nonlinear Kerr switching analysis

As schematically shown in Fig. 3a, the intense pump-probe analysis is utilized to characterize the TPA-free nonlinear all-optical Kerr switching in the Si-rich SiN_x_ micro-ring, as performed by using a single-mode pulsed data stream with a pulsewidth of 80 ps at 1549.1 nm shown in Figs. 3b and 3c. The pulsed data-stream based optical pump and the continuous-wave (CW) optical probe at deviated wavelengths are concurrently coupled into the Si-rich SiN_x_ waveguide, as illustrated in Fig. 3a. The nonlinear Kerr switching induced all-optical cross-amplitude modulation of the RZ-OOK data-stream is demonstrated under high-power pumping. As shown in Fig. 3d, the notched transmission can be spectrally red-shifted by the intensive pumping induced Kerr effect in the micro-ring. By coinciding the wavelength of pumping data stream with one notched dip as illustrated in Fig. 3e, two probes with their wavelengths at original and shifted transmission dips can be cross-amplitude modulated to provide the wavelength converted data stream with preserved and converted signs, respectively. This performs the ultrafast all-optical data format conversion with the Kerr effect induced instant increment of nonlinear refractive index at probe wavelengths.

**Figure 4:**
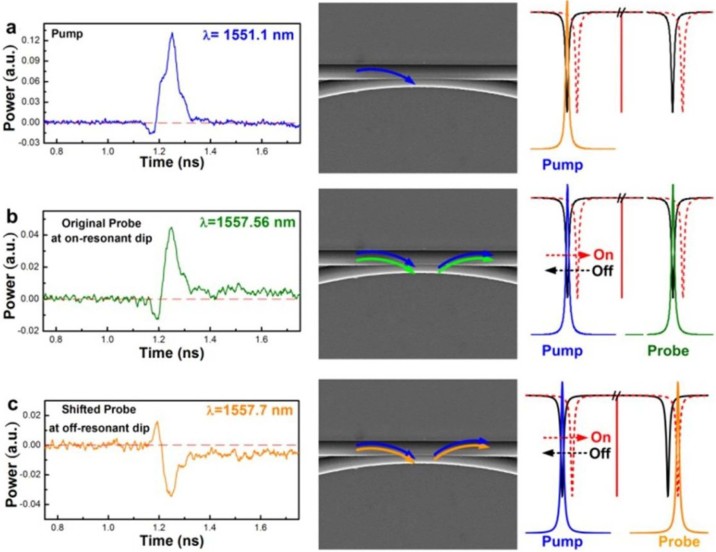
The sole “on-bit” RZ-OOK data shape responses in time-domain. The time-domain responses of a sole “on-bit” RZ-OOK data shape taken from (a) the optical pump RZ-OOK data-stream, (b) the modulated probe signal at the wavelength of the on-resonant dip without nonlinear Kerr switching, and (c) the inverted modulated probe signal at the wavelength of the off-resonant dip under nonlinear Kerr switching.

Figure 4 interprets the time-domain traces of a single bit shape for the optical pump at one notched wavelength, the sign preserved and inverted probes at wavelengths of the next on-resonant dip and the off-resonant dip (with a wavelength spacing of only 0.13 nm), respectively. As expected, the ultrafast Kerr effect of Si-QDs doped Si-rich SiN_x_ results in a transient refractive index change to spectrally shift the transmittance notch of the throughput of the bus/ring waveguide, which then induces the cross-wavelength amplitude modulation of the probe signal to demonstrate the wavelength-converted and signal-inverted data with a bit rate of up to 12 Gbit/s. The modulated data stream carried by the probe signal is identical with that carried by the optical pump. The response time of the nonlinear Kerr effect in the Si-QDs doped Si-rich SiN_x_ is in sub-picosecond regime^37^. Therefore, the modulation bandwidth of the SiN_x_ based Kerr switching is mainly dominated by the reciprocal photon lifetime of the ring resonator. From the observed quality factor at the resonant dip frequency of *ν_0_*, the limitation of modulation speed can be estimated by the photon lifetime of *τ_p_* = (*Q*/2π*ν_0_*)^−1^ inside the ring. With *Q* = 1.1 × 10^4^ at ~1550 nm, the photon lifetime of ~9 ps corresponds to a maximal modulation speed of higher than 100 Gbit/s.

With the notched wavelength shift of 0.13 nm induced under a peak intensity of *I_r_* = *P_r_*/*A_eff_* = 1.013 × 10^−9^ W/cm^2^ inside the micro-ring, the nonlinear refractive index of the Si-rich SiN_x_ is calculated as *n_2_* = *n_g_*/*I_r_* = 2.17 × 10^−13^ cm^2^/W, which is already one and two orders of magnitude larger than those of the bulk Si and the stoichiometric Si_3_N_4_, respectively^22^,^23^. The increase of *n_2_* can be attributed to the strong quantum confinement effect originated from the buried Si-QDs in the Si-rich SiN_x_ host matrix^38^. The significantly increased oscillation strength between the excitons in the Si-QDs eventually leads to the reduction on Bohr radius and enhances the third-order nonlinear susceptibility. In fact, the third-order nonlinear susceptibility is inversely proportional to the sixth power Bohr radius^39^. These Si-QDs buried in the Si-rich SiN_x_ effectively results in a huge enhancement on the nonlinear refractive index, thus leading to an efficient cross-wavelength conversion and inversion of optical data stream in the Si-rich SiN_x_ micro-ring.

### Cross-wavelength all optical data conversion

**Figure 5:**
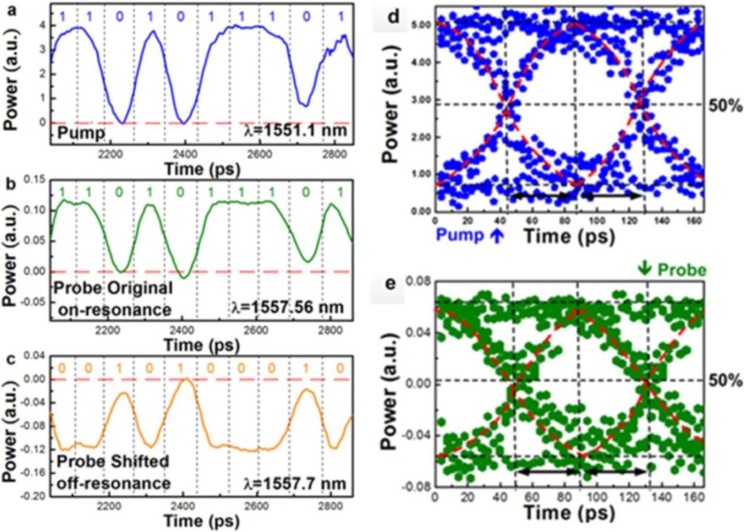
The eye-patterns and eye diagrams of the 12 Gbit//s NRZ-OOK data-streams. Left: the patterns of the 12 Gbit/s NRZ-OOK data-streams measured from the ports of (a) the optical pump input at λ = 1551.1 nm, (b) the probe output at on-resonant dip wavelength of 1557.56 nm and (c) the probe output at off-resonant dip wavelength of 1557.7 nm. Right: the eye diagrams of (d) the detected 12 Gbit/s NRZ-OOK data-stream which is employed to directly modulate the optical pump and (e) convert wavelength to the probe beam through the cross-amplitude modulation via the Si-rich SiN_x_ micro-ring based nonlinear optical Kerr switch.

To enable the Si-rich SiN_x_ micro-ring based all-optical data converter in the practical optical communication network, the Figs. 5a, 5b and 5c show the 12-Gbit/s NRZ-OOK time-domain traces of the pump data-stream, the probe data-streams obtained at wavelengths of the on-resonant and off-resonant dips, respectively. At on-resonant dip wavelength, the probe beam can be directly cross-wavelength amplitude modulated with a preserved sign as same as the original pump data-stream. In contrast, the sign-inverted probe data-stream is obtained when slightly red-shifting the probe wavelength to the off-resonant dip from the on-resonant dip by only 0.13 nm. During the interaction, the group refractive index of the micro-ring is transiently increased by the nonlinear Kerr switching effect. This instantly red-shifts the notched resonant dip from the on-resonant to the off-resonant wavelength, providing the sign inversion of the cross-wavelength amplitude modulated probe data at the off-resonant wavelength due to the inverse change of transmittance.

Figure 5 also shows the eye diagrams of the 12-Gbit/s NRZ-OOK the pump and probe data streams obtained at the Si-rich SiN_x_ bus waveguide output. The received signal-to-noise ratio (SNR) of the original pump and the cross-wavelength amplitude modulated probe data streams are 11.8 and 5.32 dB, respectively, accompanied with a degrading penalty of 6.48 dB in between. The peak-to-peak timing jitter degrades from 12.6 to 21.4 ps after cross-wavelength all-optical data conversion. Such a small degradation on the SNR shows that the Si-rich SiN_x_ micro-ring with strong Kerr effect is capable of serving as the cross-wavelength all-optical data converter and inverter for the next-generation optical interconnect applications.

## Discussion

The PECVD grown Si-rich SiN_x_ with excessive Si density of 23.4% is employed to fabricate the rib-type bus and micro-ring waveguides with insertion loss of 3 dB/facet, which exhibits dense crystalline Si-QDs buried in the Si-rich SiN_x_ to enhance ultrafast nonlinear Kerr effect, thus enabling a transient nonlinear refractive index change to achieve cross-wavelength all-optical data conversion at telecommunication wavelengths. The Si-rich SiN_x_ micro-ring based all-optical Kerr switch has been demonstrated for wavelength and format conversions of incoming optical data-stream at up to 12 Gbit/s. The presence of Si-QDs in the Si-rich SiN_x_ host matrix results in a strong quantum confinement effect to cause large optical nonlinearity. The Si-rich SiN_x_ micro-ring with *Q* = 1.1 × 10^4^ further enhances the ultrafast nonlinear Kerr effect due to the optical field enhancement of the incoming optical data-stream at its resonant wavelength. The input optical data stream instantly modifies the nonlinear refractive index of the Si-rich SiN_x_ to cause the red-shift of resonant notch, thus providing a high-speed all-optical data conversion with either preserved or inverted format via the cross-wavelength amplitude modulation effect. The nonlinear refractive index of the Si-rich SiN_x_ as high as 2.17 × 10^−13^ cm^2^/W is calculated from the wavelength red-shift of the resonance dip, which is two orders of magnitude larger than that of bulk Si or stoichiometric Si_3_N_4_. The ultrafast impulse on-off keying response as short as 83 ps at another on- or off-resonant probe wavelength is observed in the Si-rich SiN_x_ micro-ring waveguide. The eye-opening diagrams of incoming pump and converted probe data reveal small SNR degradation from 11.8 to 5.32 dB after conversion. Such a small degradation on the SNR ensures that the Si-rich SiN_x_ micro-ring is an easily applicable nonlinear optical unit, particularly suitable for all-optical cross-wavelength conversion and data-format inversion in the fiber-optic communication systems and the Si based photonic interconnection networks on chip.

## Methods

### Fabrication of Si-rich SiN_x_ all-optical Kerr switching waveguide

The Si-rich SiN_x_ channel/micro-ring based all-optical Kerr switch was fabricated by using a SiO_2_/Si-rich SiN_x_/SiO_2_ sandwiched structure on Si synthesized by plasma enhanced chemical vapor deposition (PECVD). The synthesis of the Si-rich SiN_x_ in PECVD used an argon diluted silane (90% Ar + 10% SiH_4_) mixed with nitrous (NH_3_) gaseous recipe at substrate temperature of 350°C and a RF plasma power of 100 W. During the PECVD growth, the chamber pressure is remained at 134 Pa. The fluence ratio of [SiH_4_]/[NH_3_] is as high as 0.9, which facilitates the growth of the Si-rich SiN_x_ at low RF plasma power regime. Afterwards, the waveguide pattern was defined by using E-beam lithography, the RIBE process with an optimized recipe on the fluence ratio of the CHF_3_ + O_2_ gaseous mixture was used to remove the unpatterned Si-rich SiN_x_ layer deposited on the SiO_2_ covered Si substrate. After removing the Cr mask, a 2-μm thick SiO_2_ upper cladding layer was deposited by PECVD at a standard recipe.

### Analytic setup for cross-wavelength data conversion and format inversion

In the communication testing bench, a CW optical probe signal and a high-power optical pump data stream are concurrently coupled into the Si-rich SiN_x_ channel waveguide through a 50/50 coupler and a lensed fiber. To generate a high-power optical data-stream as the pump beam, a Mach-Zehnder modulator (MZM, JDSU, 10024180) is introduced to externally modulated the pump source served by a tunable laser (TL, HP, 8168F) at a wavelength (1551.1 nm) shorter than that of the probe beam (1557.56 nm). The MZM is encoded by the RZ-OOK data-stream at 12 Gbit/s from an arbitrary waveform generator (AWG, Tektronix, 7122B). The optical pump beam with a RZ-OOK format is further pre-amplified by an erbium-doped fiber amplifier (EDFA, JDSU, OAB1552 + 20FA6), and subsequently filtered by an optical bandpass filter (OBPF, SANTEC, OTF-910) with a 3-dB linewidth of only 0.4 nm to suppress the additional amplified spontaneous emission (ASE) noise added during pre-amplification. Then, a second set of EDFA (SDO, EFAH1B111NC02) and OBPF (JDS, TB1500B) are employed to boost-amplify the peak power of the optical pump power as high as P_peak_ = 3 W (equivalent to P_avg_ = 16 dBm at 12 Gbit/s.) At the probe part, a CW single mode tunable laser (TL, Agilent, 8164A) at a wavelength slightly longer than the pump beam is employed. The probe power is conditionally amplified by another EDFA. At the receiving end, another OBPF with its central wavelength set coincident with that of the probe beam is used to separate the cross-amplitude modulated probe signal from the remaining pump signal. A photo diode (PD, Notel, pp-10G) is used to detect the cross-amplitude modulated RZ-OOK data-stream carried by the probe beam, and the output is monitored by a digital sampling oscilloscope (DSO, Agilent, 86100A + 86109B) for eye-pattern analysis.

## Author Contributions

G.-R.L. proposed the concept. G.-R.L., S.P.S., C.L.W. and B.J.H. designed the experiment. S.P.S., C.L.W. and B.J.H. fabricated the Si-rich SiN_x_ micro-ring waveguide, and established the analytic setup for cross-wavelength data conversion and format inversion. S.P.S., C.L.W. and B.J.H. carried out the experimental data. H.Y.W., C.T.T. and Y.C.C. generated the NRZ-OOK signals and measured the eye diagrams of the transmitted signals. S.P.S., C.L.W., B.J.H. and G.-R.L. analyzed and simulated the data. G.R.L., S.P.S., C.L.W. and Y.H.L. contributed to the writing of the manuscript.

## Figures and Tables

**Table 1:** The structural and material parameters of the Si-rich SiN_x_ micro-ring resonator

*M*	*α_r_* (μm^−1^)	*κ*′ (μm^−1^)	*l_i_* (μm)	*L_r_* (μm)	*θ* (rad)
2.6	7 × 10^−4^	0.0073	32	100π	2π
